# Janerin Induces Cell Cycle Arrest at the G2/M Phase and Promotes Apoptosis Involving the MAPK Pathway in THP-1, Leukemic Cell Line

**DOI:** 10.3390/molecules26247555

**Published:** 2021-12-13

**Authors:** Mohammad Z. Ahmed, Fahd A. Nasr, Wajhul Qamar, Omar M. Noman, Javed Masood Khan, Abdullah A. Al-Mishari, Ali S. Alqahtani

**Affiliations:** 1 Department of Pharmacognosy, College of Pharmacy, King Saud University, Riyadh 11451, Saudi Arabia; mahmed4@ksu.edu.sa (M.Z.A.); onoman@ksu.edu.sa (O.M.N.); aalmshari@ksu.edu.sa (A.A.A.-M.); 2 Department of Pharmacology and Toxicology, College of Pharmacy, King Saud University, Riyadh 11451, Saudi Arabia; wqidris@ksu.edu.sa; 3 Department of Food Science and Nutrition, College of Food and Agricultural Sciences, King Saud University, Riyadh 11451, Saudi Arabia; jmkhan@ksu.edu.sa

**Keywords:** *Centaurothamnus maximus*, janerin, Bax/Bcl-2, caspase

## Abstract

Janerin is a cytotoxic sesquiterpene lactone that has been isolated and characterized from different species of the *Centaurea* genus. In this study, janerin was isolated form *Centaurothamnus maximus*, and its cytotoxic molecular mechanism was studied in THP-1 human leukemic cells. Janerin inhibited the proliferation of THP-1 cells in a dose-dependent manner. Janerin caused the cell cycle arrest at the G2/M phase by decreasing the CDK1/Cyclin-B complex. Subsequently, we found that janerin promoted THP-1 cell death through apoptosis as indicated by flow cytometry. Moreover, apoptosis induction was confirmed by the upregulation of Bax, cleaved PARP-1, and cleaved caspase 3 and the downregulation of an anti-apoptotic Bcl-2 biomarker. In addition, immunoblotting indicated a dose dependent upregulation of P38-MAPK and ERK1/2 phosphorylation during janerin treatment. In conclusion, we have demonstrated for the first time that janerin may be capable of inducing cell cycle arrest and apoptosis through the MAPK pathway, which would be one of the mechanisms underlying its anticancer activity. As a result, janerin has the potential to be used as a therapeutic agent for leukemia.

## 1. Introduction

Acute myeloid leukemia (AML) is a kind of hematopoietic malignancy marked by the abnormal clonal expansion of myeloid blast cells in bone marrow and peripheral blood. Although the majority of cases occur in adults, it is the second most common form of leukemia in both adults and children, and it has the lowest survival rate among all forms of leukemia [1]. Different therapies have been employed for AML including radiation therapy and chemotherapy [2]. Additionally, the current understanding of AML pathophysiology has resulted in the approval of several medicines for various symptoms of AML [3]. However, all these choices still cause numerous side effects in a broad range of body cells [4]. Natural products from medicinal plants represent an alternative choice with minimum side effects.

Over several decades, natural compounds research in the field of cancer has led to the discovery of many anticancer drugs [5]. Some of the anticancer drugs that have been directly derived from herbs and natural plants are vinblastine, camptothecin, and paclitaxel [6]. *Centaurothamnus maximus* is an plant, native to the Arabian Peninsula; it is a leafy shrub and belongs to the Asteraceae family [7]. Previous phytochemical studies on *C. maximus* have revealed the presence of numerous phytoconstituents such as sesquiterpene lactones, elemanolides, germacranolides, and guaianolides [8]. Among these phytoconstituents, sesquiterpene lactones were some of the most prominent constituents in this species [8,9,10]. Sesquiterpene lactones are a kind of sesquiterpene with a lactone ring; they have a variety of bioactivities, the most notable of which is antineoplastic activity, which has been demonstrated in multiple in vitro and in vivo studies [11].

Janerin is a cytotoxic sesquiterpene lactone that has been previously isolated from *C. maximus* [12]. In their original work, Muhammad et al. (2003) documented that janerin exerted a potent cytotoxic effect on different cancer cells. In addition, janerin isolated from *Rhaponticum repens* showed a promising inhibitory effect on HUVEC cell migration [13]. In the current work, the impact and action mechanism of janerin were investigated in THP-1 human leukemic cells, for the first time. Our findings clearly demonstrated that the janerin arrested the THP-1 cells at the G2/M phase and induced apoptosis through the activation of the MAPK pathway.

## 2. Results

### 2.1. Identification of the Components

**Figure 1:**
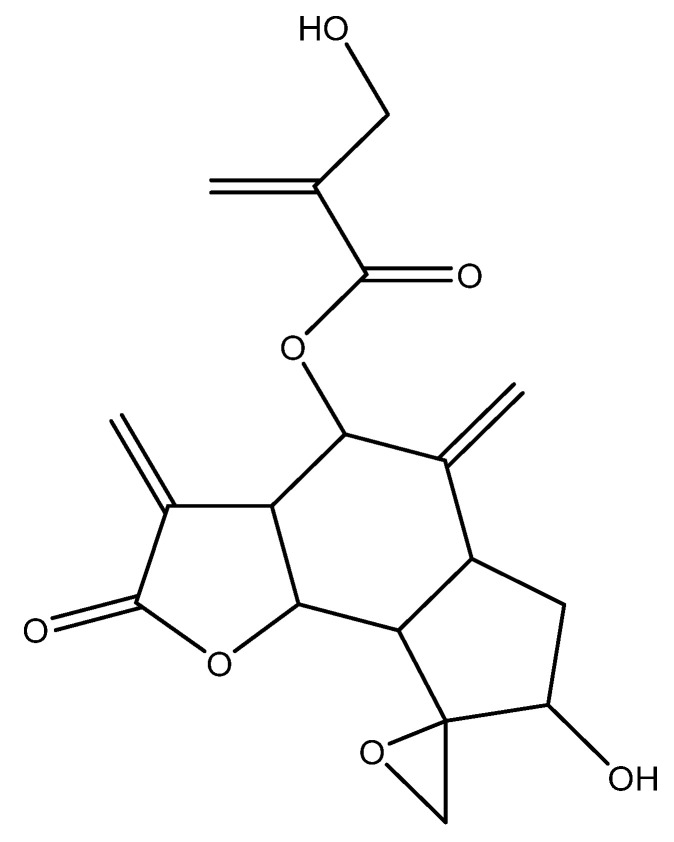
The chemical structure of janerin, which was isolated from the chloroform fraction of *C. maximus*.

*Centaurothamnus maximus* (CMA-1) was isolated as white crystals. Its mass spectral data suggested a molecular formula of C_19_H_22_O_7_. The ^1^H NMR (700 MHz, DMSO) δ 6.18 (s, 3H), 5.97 (s, 3H), 5.91 (s, 3H), 5.48 (s, 3H), 5.31 (s, 3H), 5.15 (d, *J* = 10.8 Hz, 6H), 5.06 (s, 3H), 5.03 (s, 3H), 4.87 (s, 2H), 4.71 (s, 3H), 4.18 (s, 6H), 4.07 (d, *J* = 11.1 Hz, 3H), 3.91 (s, 3H), 3.72 (d, *J* = 11.2 Hz, 3H), 3.36 (s, 87H), 3.07 (s, 3H), 2.58 (s, 2H), 2.51 (s, 12H), 2.30 (d, *J* = 14.8 Hz, 6H), 2.24 (s, 2H). ^13^C NMR (176 MHz, DMSO) δ 169.05 (s), 165.41 (s), 144.38 (s), 141.38 (s), 138.51 (s), 124.60 (s), 121.05 (s), 116.90 (s), 84.14 (s), 77.04 (s), 75.46 (s), 73.94 (s), 60.04 (s), 58.42 (s), 50.06 (s), 47.63 (s), 46.04 (s), 34.73 (s). Completion of the NMR data interpretation process, which included correlation spectroscopy (COSY), heteronuclear single quantum coherence (HSQC), and heteronuclear multiple bond correlation (HMBC) studies, led to the identification of CMA-1 as janerin (Figure 1).

### 2.2. Janerin Suppresses THP-1 Cell Growth In Vitro

**Figure 2:**
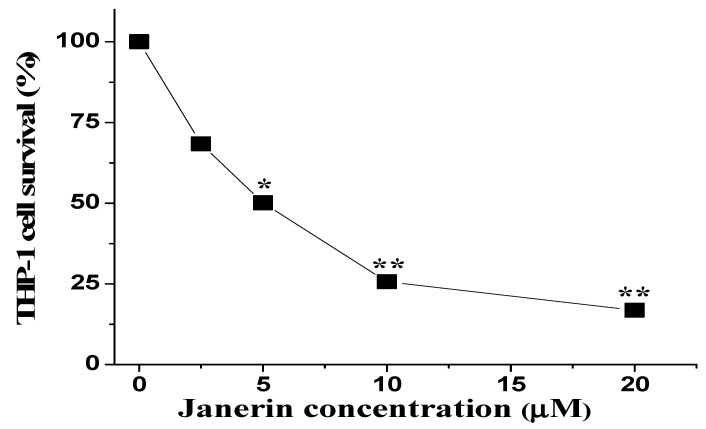
Janerin suppresses the proliferation of THP-1 cells. The cells were treated with increasing concentrations of janerin for 24 h and cell viability was determined by MTT assay. Cell viability was expressed as a %, compared to the control. * *p* < 0.05 and ** *p* < 0.01 versus control.

Human monocytic leukemia cells (THP-1) were treated with varied dosages of janerin to determine its cytotoxicity. The results showed that janerin dramatically reduced the growth of THP-1 cells in a dose-dependent manner (Figure 2). The reported IC_50_ value (half inhibitory concentration) of THP-1 cell growth was 5 µM. Based on previous IC_50_ values, three different concentrations (1/2 IC_50_, IC_50_, and 2 × IC_50_) were selected for the further experiments.

### 2.3. Janerin Causes Accumulation of Cells in the G2/M Phase

**Figure 3:**
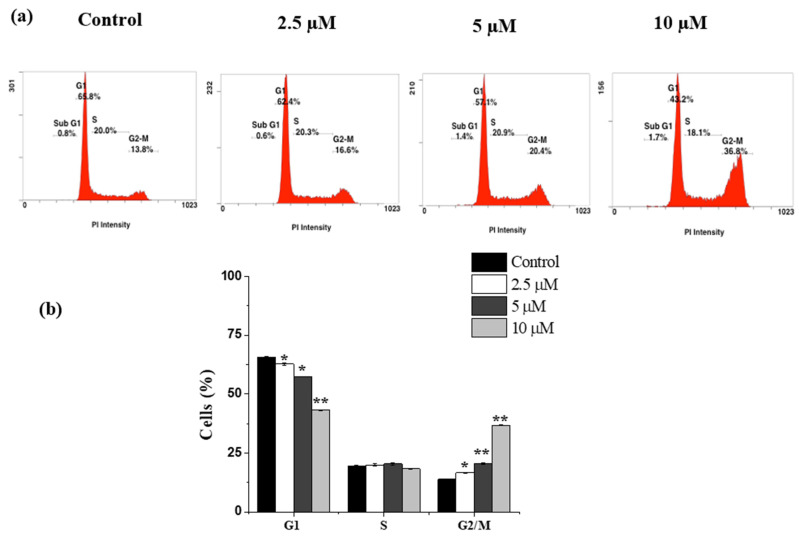
Janerin induces G2/M cell cycle arrest in THP-1 cells. The THP-1 cells were incubated with or without different concentrations of janerin (2.5, 5, and 10 µM) for 24 h. The cells harvested after 24 h were subjected to cell cycle analysis. (**a**) DNA contents of janerin-treated THP-1 cells demonstrated an increase in the proportion of the G2/M phase compared to that of untreated cells. (**b**) The percentage of cells in each phase was calculated and plotted into a histogram. * *p* < 0.05 and ** *p* < 0.01.

Cell cycle analysis was used to investigate the inhibition of THP-1 cell growth. THP-1 cells were treated with different concentrations of janerin (2.5, 5, and 10 µM) over 24 h, and then flow cytometry cell cycle analysis was performed. As shown in Figure 3, the percentage of G2/M phase cells was higher in the treated group than in the control group. In comparison to the control group, the percentage of the G2/M phase cell population (13.9 ± 0.14%) was increased to 16.65 ± 0.1%, 20.6 ± 0.28%, and 36.9 ± 0.14%, at concentrations of 2.5, 5, and 10 µM, respectively (Figure 3).

**Figure 4:**
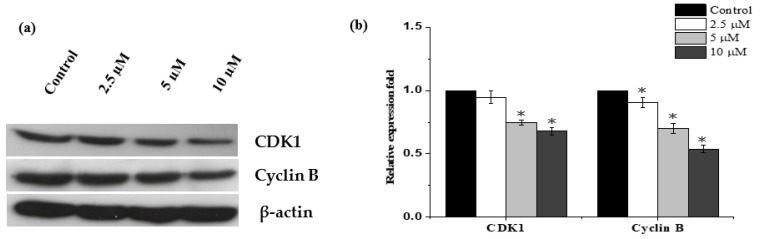
Janerin induces G2/M cell cycle arrest in THP-1 cells through downregulation of CDK1 and cyclin B. The THP-1 cells were incubated with or without different concentrations of janerin (2.5, 5, and 10 µM) and incubated for 24 h. (**a**) Janerin-treated THP-1 cells demonstrated a greater downregulation of CDK1 and cyclin B in comparison to that of untreated cells. (**b**) The protein bands on the blots were measured relative to the control. * *p* < 0.05 compared to control.

To further study how janerin causes G2/M arrest, we looked at how it affected mitotic regulatory proteins including cyclin B and CDK-1, which play an important role in inducing mitosis. We found that janerin significantly reduced cyclin B1 and CDK-1 levels in a dose-dependent manner (Figure 4), confirming that the lowering of these proteins caused a cell cycle arrest at the G2/M phase.

### 2.4. Janerin Causes Apoptosis in THP-1 Cells

**Figure 5:**
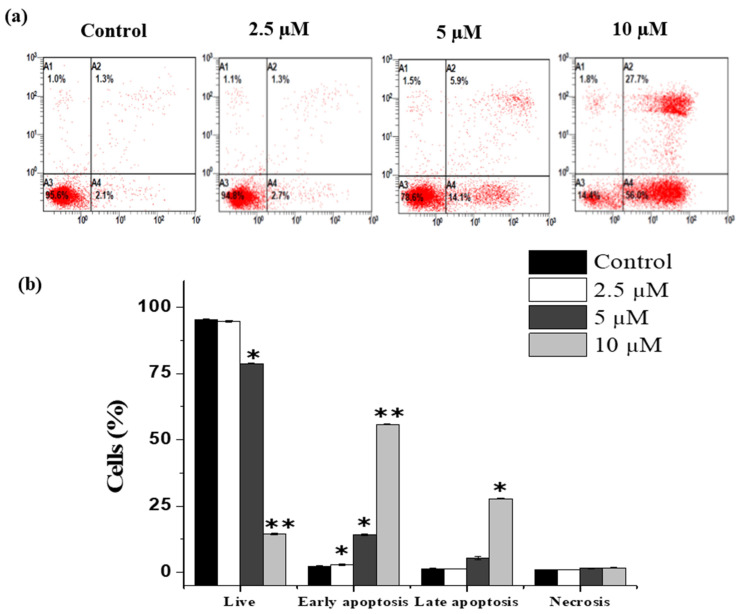
Apoptosis rate increment, as affected by the increase in janerin concentration in THP-1 cells. The THP-1 cells were treated with different concentrations of janerin (2.5, 5, and 10 µM) and incubated for 24 h and were compared with untreated cells. (**a**) (A1) The upper left quadrant indicates the dead cells, (A2) the upper right quadrant represents the late apoptotic cells, (A3) the lower right quadrant signifies the early apoptosis, and (A4) the lower left quadrant shows the viable cells. (**b**) Bar chart indicates the percentage of cells in each stage. (* Statistically significant difference from the control group, * *p* < 0.05, ** *p* < 0.01).

Flow cytometry was then carried out to determine the percentage of apoptotic cells using the well-known Annexin V/PI dual labelling method. Within 24 h of treatment, flow cytometry analysis of the treated THP-1 cells showed that the cells had shifted from viable to early apoptotic phase. The percentage of early apoptotic cells increased to 2.95 ± 0.2%, 14.2 ± 0.28%, and 55.85 ± 0.3% as compared to the healthy control cells, where it was 2.3 ± 0.2% as shown in (Figure 5). Furthermore, as janerin concentrations increased, the percentage of late apoptotic cells increased as well, although necrotic cell death did not increase (Figure 5). These results clearly demonstrated the ability of janerin to induce the apoptotic death of THP-1cells.

### 2.5. Janerin Modulates Bax and Bcl-2 Expression

**Figure 6:**
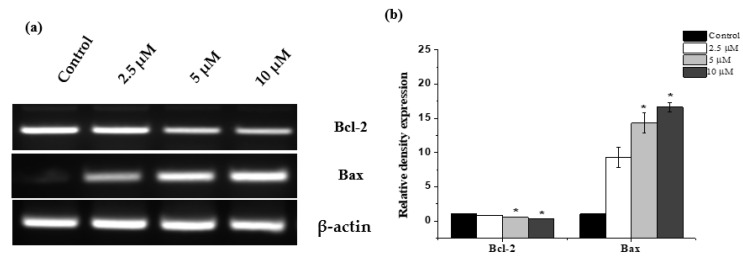
RT-PCR analysis of Bcl-2 and Bax gene expression in THP-1 cells treated with different concentrations of janerin for 24 h. (**a**) Bands represent the PCR products for Bax, Bcl2, and β-actin, which were produced from the total cellular RNA isolated from untreated and janerin-treated THP-1 cells. (**b**) The bar-graphs represent mean ± SD of mRNA expression for Bcl-2 and Bax relative to control. (* Statistically significant difference from the control group, * *p* < 0.05).

Next, the expression levels of Bax and Bcl-2 were assessed in THP-1 cells after 24 h of janerin treatment. A significant upregulation in the expression of Bax was observed (Figure 6) with the increasing janerin concentration (9.3 ± 1.2, 14.3 ± 1.45, and 16.6 ± 0.7-fold, respectively). In the same manner, the expression level of Bcl-2 was decreased significantly with the increasing concentration of janerin in the period of 24 h (downregulated to 0.8 ± 0.06, 0.53 ± 0.03, and 0.3 ± 0.01-fold, respectively) (Figure 6).

### 2.6. Western Blot Analysis of Apoptosis-Related Proteins Modulated by Janerin

**Figure 7:**
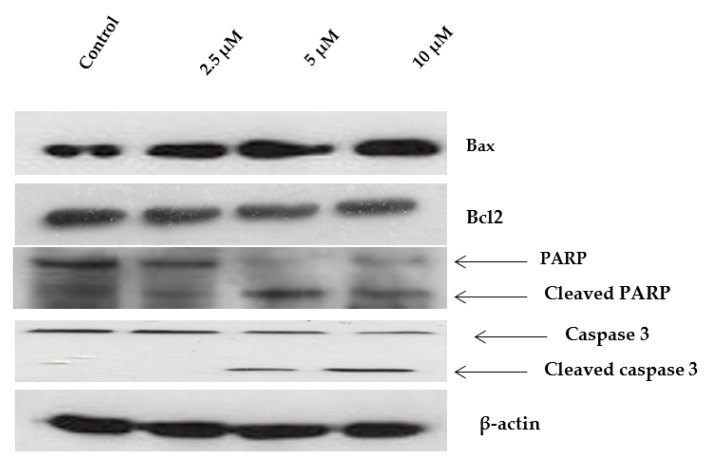
Effect of janerin on the protein expression of Bax, Bcl2, PARP, and caspase 3 in THP-1 cells. Western blot analysis of protein expression was carried out after the janerin treatment at different concentrations. The β-actin blot was used as an internal control.

The apoptosis-related proteins Bax, Bcl-2, PARP, and caspase 3 were further analyzed using Western blot analysis to assess the aforementioned apoptotic activity, which was produced by janerin. The results indicate that janerin markedly increased the expression of apoptotic protein Bax and simultaneously decreased anti-apoptotic protein Bcl-2 expression in a concentration-dependent manner (Figure 7). The cleaved products of PARP and caspase 3 were consistently detected in the treatment with increasing concentrations of janerin (Figure 7).

### 2.7. Janerin-Induced Apoptosis via Activation of p38MAPK

**Figure 8:**
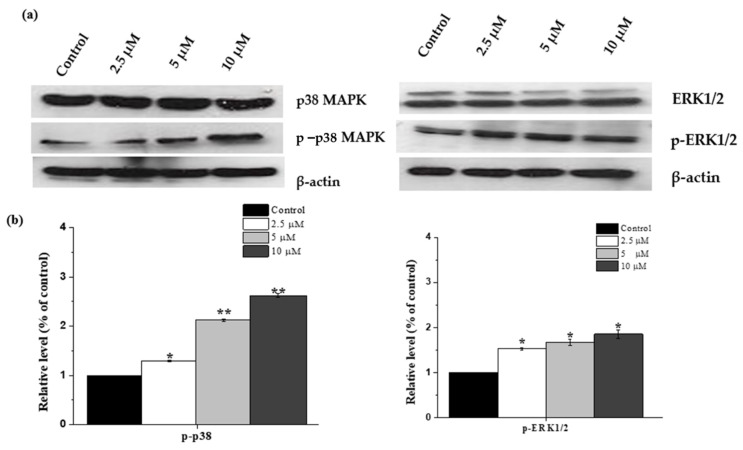
Effect of janerin treatment on p38 and ERK activation. (**a**) THP-1 cells were treated with different concentrations of janerin for 24 h, and phosphorylated p38 MAPK and ERK1/2 were detected in janerin-treated as well as untreated THP-1 cells using specific antibodies. (**b**) Quantitative data of p-p38 MAPK and p-ERK1/2 protein levels were adjusted for β-actin protein levels and expressed as fold relative to control. The data are presented as a mean standard deviation (*n* = 3). * *p* < 0.05, ** *p* < 0.01.

Following previous findings, which clearly demonstrated the occurrence of apoptosis in THP-1 cells by janerin, we explored whether the mitogen-activated protein kinase (MAPK) pathways might be involved in janerin’s apoptotic action. As shown in Figure 8, our results indicate that janerin increased the phosphorylation of p38 MAP- kinase (p38 MAPK) and extracellular signal-regulated kinase (ERK1/2) in a dose-dependent manner.

## 3. Discussion

Several medicinal plants have been shown to be an effective natural source of anti-leukemic medicines, and further research is being conducted in this area [14]. Previously, janerin was reported to effectively inhibit the proliferation of epidermoid (KB), ductal (BT-549), malignant melanoma (SK-MEL), and ovarian (SK-OV-3) cancer cells [12]. In this study, we found that janerin possessed anti-leukemic capabilities by inhibiting the proliferation of THP-1 human leukemic cells. Furthermore, our findings demonstrated that janerin-induced cell death was the result of cell accumulation in the G2/M phase and apoptosis induction via the MAPK pathway. According to MTT assay data, THP-1 cells treated with janerin exhibit a dose-dependent decrease in their ability to multiply, which may be due to cell death initiation. Based on the calculated IC_50_, the concentration range of janerin (2.5, 5, and 10 µM) was chosen for further assessment.

The target of cancer treatment is the cell cycle arrest, and apoptosis induction may be the most important goal in contemporary cancer treatment [15,16]. In order to further understand the reported antiproliferative action of janerin, cell cycle analysis was carried out. We found that janerin treatment led to the accumulation of the cells in the G2/M phase. Indeed, when the cell’s DNA is damaged, cell cycle interruption occurs in this phase in order to provide the time for repairing the damaged DNA [17]. Numerous regulatory proteins including the CDK1/cyclin B complex are involved in this checkpoint phase and control cell transit through the G2/M phase [18,19]. Accordingly, the expression of the CDK1/cyclin B complex was evaluated to clarify its involvement in cell cycle arrest mediated by janerin. Consequently, the levels of CDK1 and cyclin B were decreased after treatment with janerin, providing further evidence for suggesting that janerin might arrest the cell cycle at the G2/M phase. Our findings are in line with several previous studies that documented the downregulation of the CDK1/cyclin B complex when the cell cycle is arrested at the G2/M phase [20,21]. Furthermore, the persistent arrest of the cell cycle at this phase is usually followed by the apoptotic process [22]. In fact, most chemotherapeutic agents isolated from natural sources exert their effects by inducing the apoptosis of cancer cells [23,24]. Therefore, we next explored whether janerin might be able to initiate the apoptotic process. The externalization of phosphatidylserine (PS) is an important biochemical change during the early stages of apoptosis, which can be easily detected using Annexin V/FITC and PI dual staining assay with the help of flow cytometry [25]. Additionally, an alteration in the gene expression of Bcl-2 family members, in particular, pro-apoptotic Bax and anti-apoptotic Bcl-2 genes, is considered a molecular marker associated with the initiation of apoptosis [26,27,28]. The data presented here indicated that the percentage of early and late apoptotic cells was increased by exposing the increasing concentration of janerin for 24 h. Janerin-induced apoptosis through upregulation of pro-apoptotic Bax and downregulation of anti-apoptotic Bcl-2 was also demonstrated in this study. This shift in the Bax/Bcl-2 ratio followed by PARP cleavage and caspase-3 activation could provide additional molecular evidence that janerin triggered apoptosis in THP-1 cells.

The MAPK pathway is well known to be involved in both cell proliferation and apoptosis in response to cellular stress [29,30]. Hence, members of the MAPK pathway such as p38 MAPK and ERK were studied further to elucidate the involvement of the MAPK pathway in janerin apoptotic action. Our findings indicated a remarkable increase in the phosphorylation of ERK1/2 and p38 after janerin treatment, and these findings are consistent with the involvement of both ERK and p38 in the activation of apoptosis. Our results here are in line with previous findings, indicating that the 1-cinnamoyltrichilinin compound, isolated from *Melia azedarach*, caused an increase in the phosphorylation level of p38-MAPK in HL-60 leukemia cells [31]. Moreover, several dietary phytochemicals have been also reported to cause apoptosis by activating the MAPK pathway in various cancer cells [32,33].

## 4. Materials and Methods

### 4.1. Plant Collection, Extraction and Fractionation

Aerial parts of *C. maximus* were collected from the Al-Baha region, Saudi Arabia, in March, 2019. They were authenticated by Prof. Mothana in the Pharmacognosy Department, College of Pharmacy, King Saud University, Saudi Arabia. Using a Soxhlet apparatus, dried powder (500 g) from the aerial portions was extracted with ethanol (3 L). A rotary evaporator was employed to dry up the liquid extract, and approximately 57 g of the crude semi-solid ethanolic extract was collected. Subsequently, ethanolic crude extract was successively distributed to different polar solvents, i.e., *n*-hexane, chloroform, and n-butanol, to obtain 3.5, 17, and 23 g of dried fractions, respectively.

### 4.2. Compound Isolation and Identification

Separation of CMA-1 (23 mg) was performed using silica gel column chromatography as described previously [12]. In brief, the chloroform fraction (5 g) was added to the top of a silica gel packed column (72 g, 80 cm × 3 cm). Elution began with 3% methanol: chloroform, and the polarity was increased with methanol in gradient mode of elution, giving 13 fractions (20 mL each). Based on TLC behavior, the same fractions were collected and pooled together to yield seven main fractions. Since the cytotoxic activity recovered in fraction D (47 mg), fraction D then eluted with 30% chloroform: methanol, which was re-chromatographed on a silica gel column (7.2 g, 60 cm × 1 cm) using a gradient mixture consisting of chloroform and methanol as an elution solution. According to their TLC behavior, the same fractions were collected to give five sub-fractions. Sub-fraction 2, eluted with 50% methanol: chloroform, yielded compound CMA-1 (23 mg). Several spectroscopic techniques including 1D and 2D-NMR were applied to the determination of chemical structure.

### 4.3. MTT Assay

THP-1 cells in logarithmic growth phase were seeded at a density of 1 × 10^5^ cells per well on a 24-well plate and treated with various doses of janerin (1.25, 2.5, 5, 10, and 20 µM) for 24 h to assess cytotoxicity [34]. After exposing the cells for 24 h, the MTT solution (5 mg/mL MTT powder in 1× PBS) was applied to the cells and incubated for 2 h at 37 °C in a 5% CO_2_ incubator. During this period of incubation, MTT was reduced to purple formazan only by viable cells, which was further solubilized by DMSO. The absorbance was measured at 570 nm wavelength (BioTek Instruments Inc., Swindon, UK). The number of viable cells was calculated according to the equation of %V = A − Ao / Ac − Ao × 100, where V is the viability of cells, A is the absorbance of the treated cells, Ao is the absorbance of blank, and Ac is the absorbance of control.

### 4.4. Cell Cycle Analysis

THP-1 cells were exposed to different concentrations (2.5, 5, and 10 µM) of janerin for 24 h, and a cell cycle assay was performed as described previously [35]. Both janerin-treated and untreated cells were collected after 24 h exposure, washed twice with 1× PBS, and subsequently fixed with 70% ethanol (pre-cooled) with gentle vortexing and kept for 4 h at 4 °C. After 4 h of incubation, the fixed cells were precipitated by centrifuging at 1200 rpm for 10 min to get rid of the ethanol. Subsequently, cells were washed twice with 1× PBS, re-suspended in 500 mL of 1× PBS with 0.1 mg/mL Ribonuclease A, and incubated at room temperature for 30 min. After incubating, 0.01 mg/mL of PI was added to each tube, at which point the tube was ready to detect the fluorescence of the PI-DNA complex at different stages of the cell cycle, using Flow Cytometry (Beckman Coulter) and CXP software V. 3.0. software.

### 4.5. Determination of Apoptosis by Fluorescence-Activated Cell Sorting

The logarithmic growth phase of THP-1 cells (1 × 10^6^) were plated on a 12-well plate and treated with different concentrations of janerin (2.5, 5, and 10 μM); control cells were treated with 0.01% DMSO. After incubating for 24 h, an Annexin V/PI staining Kit was employed to detect apoptosis cells induced by janerin as described previously [36]. In summary, cells were harvested, washed in 1× PBS, and resuspended in 100 µL of binding buffer (contained in the kit); then 5 µL of each dye was added (Annexin FITC and PI). After 30 min, 400 µL of binding buffer was added to the labelled cells, which were subsequently examined using a FACS Scan Flow Cytometer (Cytomics FC 500; Beckman Coulter, Brea, CA, USA).

### 4.6. RT-PCR Analysis

TRIzol reagent (Invitrogen; Thermo Fisher Scientific, lnc., Waltham, MA, USA) was employed for RNA extraction from both janerin-treated and untreated THP-1 cells according to manufacturer’s protocol. Nanodrop was used to estimate total RNA amount, and 2 µg of RNA was reverse transcribed according to the manufacturer’s instructions, using Superscript IV Vilo Master mix (Invitrogen; Thermo Fisher Scientific, lnc., Waltham, MA, USA). The cDNA samples were stored at −20 °C. A semiquantitative PCR was performed to estimate Bcl-2 and Bax gene expression levels. The final volume (20 μL) of the RT-PCR mixture consisted of 2 µL of cDNA, 4 µL of 5× FIRE pol Master mix (Solis BioDyne, Tartu, Estonia), and 10 pmoles of each complementary primer specific for Bax, Bcl-2, and GAPDH as an internal control, and the rest of the volume consisted of 4 µL RNase free water. The sample was initially denatured at 95 °C for 5 min and amplified using 32 cycles of denaturation at 95 °C for 30 s, annealing at 55 °C for 60 s, and elongation at 72 °C for 60 s, followed by final elongation at 72 °C for 5 min. Thereafter, 20 μL of the PCR amplified sample was then subjected to an electrophoresis using a 1.2% agarose gel containing ethidium bromide, and the bands on the gel were photographed.

### 4.7. Western Blot Analysis

The janerin-treated and untreated cells were washed twice with 1× PBS and lysed by incubating in the lysis buffer (20 mM Tris-HCl (PH 7.5), 0.5 M NaCl, 1 mM EDTA, 1 mM EGTA, 0.25% Triton X-100, protease inhibitor mixture, 2 mM PMSF, and 1mM DTT) for 2 h on ice. The obtained lysate was clarified by centrifuging at 1300 rpm at 4 °C for 15 min. The protein content of the supernatant was measured using the Bradford protein assay (Bio-Rad, Hercules, CA, USA). Thirty micrograms of protein were resolved by SDS-PAGE using 12% gel and transferred to a PVDF membrane. The membrane was blocked with 5% BSA followed by washing with TBST and incubation with primary antibodies (1:2000) against Bax, Bcl2, P-p38, P38, and β-actin overnight at 4 °C. After removing the primary antibodies and washing the membranes three times with TBST, the membranes were incubated for 30 min at room temperature with an HRP-conjugated secondary antibody (1:4000). Then, the membrane was rinsed with TBST, and the signals were visualized with an ECL reagent and exposed to an X-ray film.

### 4.8. Statistical Analysis

Statistical analysis was performed using OriginPro 8.5 software. The signal intensities were determined by computer-assisted densitometry using Image J software. Data were shown as mean ± standard deviation. Significance was analyzed by student *t*-test. A difference was considered statistically significant when *p* < 0.05 (*); *p* < 0.01 (**); *p* < 0.001 (***).

## 5. Conclusions

In conclusion, we present for the first time abundant evidence demonstrating janerin’s possible role in triggering G2/M cell cycle arrest and apoptotic cell death in THP-1 cells. Janerin caused cell cycle arrest at the G2/M phase, as evidenced by decreased expression levels of CDK1 and cyclin B proteins. Apoptosis initiation through Bax, PARP, and caspases 3 activation was also recognized. Janerin-mediated apoptotic cell death could possibly be linked to the activating of p38/ERK molecules in the MAPK signaling pathway. These findings enhanced our understanding of the molecular pathway through which janerin exerted its anticancer activity, and which in turn could be important in the development of novel treatments for leukemia. In order to better understand the impact of janerin, further studies in an in vivo model are required to validate the obtained findings.

## Data Availability

The data presented in this study are available on request from the corresponding author.

## Sample Availability

Samples are available from the authors upon reasonable request.
